# DEVELOPMENT AND IMPLEMENTATION OF AN INTERDISCIPLINARY HONORS COURSE ON AGING

**DOI:** 10.1093/geroni/igz038.540

**Published:** 2019-11-08

**Authors:** Mary C DiBartolo

**Affiliations:** Salisbury University, Salisbury, Maryland, United States

## Abstract

With the aging of Baby Boomers, 2030 will mark the first time in U.S. history that those aged 65 and older will outnumber children. This population shift is expected to place unprecedented demands on the healthcare system in terms of both volume and complexity of care. Given these population shifts and emphasis on an interdisciplinary approach to care, a 4-credit Honors aging course was developed for Honors students in nursing and other health-related majors. Aging Reexamined, Reimagined is offered in a discussion/seminar format with limited enrollment to allow for deep reflective discourse about pertinent issues affecting older adults. Topics include physical/cognitive changes, ageism, Alzheimer’s disease, sexuality, aging in place, polypharmacy, addiction, depression, caregiving, elder justice, and end-of-life care. Guest speakers share their expertise on selected issues, otherwise students alternate leading discussions on remaining topics. There are three focused reflections on assigned experiences which include conducting a videotaped interview with a retired community-based older adult, attending a support group or senior center activity, and visiting a center to view various physical/technological adaptive aids that maintain mobility and independence in the home. There is also a culminating research paper on an issue of their choice. Student evaluations are overwhelmingly positive; comments include gaining in-depth knowledge about the unique needs of this population and the importance of healthy aging with an emphasis on a positive, inter-professional approach to care. It is incumbent upon educators to better prepare students to recognize ageist attitudes, as well as address the significant impact of this “longevity revolution”.

